# Non-invasive treatments improve patient outcomes in chronic tinnitus: a systematic review and network meta-analysis

**DOI:** 10.1016/j.bjorl.2024.101438

**Published:** 2024-05-02

**Authors:** Tingting Lu, Qingxin Wang, Ziyan Gu, Zefang Li, Zhaojun Yan

**Affiliations:** a Shandong University of Traditional Chinese Medicine, First Clinical College of Medicine, Jinan, China; b The Second People’s Hospital of Qingdao West Coast New District, Department of General Internal Medicine, Qingdao, China; c Affiliated Hospital of Shandong University of Traditional Chinese Medicine, Department of Physical and Mental Medicine, Jinan, China

**Keywords:** Non-invasive, Chronic tinnitus, Network meta-analysis, Cognitive behavioral therapy, Acoustics

## Abstract

**Objective:**

To investigate the relative effectiveness of various Non-Invasive Treatment Techniques (NITs) in chronic tinnitus management.

**Methods:**

We searched PubMed, Embase and Cochrane Library databases from the time of data construction to December 31, 2022. According to the Preferred Reporting Items for Systematic Reviews and Meta-Analyses guidelines, NITs were evaluated, including Aacceptance and commitment therapy (A), Cognitive behavioral therapy (C), Sound therapy (S), Transcranial magnetic stimulation (T), Electrical stimulation therapy (E), Virtual reality therapy (V), tinnitus Retraining therapy (R), general psychotherapy (D), and Placebo (P). The outcome indicators included the Tinnitus Handicap Inventory (THI), Tinnitus Questionnaire (TQ), Hospital Anxiety and Depression Scale-anxiety-Depression (HADS-D), Insomnia Severity Index (ISI), Visual Analogue Scales-Loudness (VAS-L), and Visual Analogue Scales-Distress (VAS-D). Statistical analysis was performed using Stata 14.0 for NMA.

**Results:**

This systematic review and meta-analysis included 22 randomized controlled trials comprising 2,354 patients. The treatment effects varied on each scale. For THI, S (86.9%) was the most effective, whereas P (6.5%) was the worst. For TQ, C (89.5%) was the most effective, whereas D (25.4%) was the worst. For HADS-D, A (82.4%) was the most effective, whereas D (9.47%) was the worst. For ISI, A (83.2%) was the most effective, whereas R (20.6%) was the worst. For VAS-L, S (73.5%) was the most effective, whereas E (18.9%) was the worst. For VAS-D, C (84.7%) was the most effective, whereas P (18.1%) was the worst.

**Conclusions:**

The combination of acoustics and cognitive behavioral therapy may be an effectively treat patients with chronic tinnitus.

**Level of evidence:**

How common is the problem? Level 2.

Is this diagnostic or monitoring test accurate? (Diagnosis) Level 1.

What will happen if we do not add a therapy? (Prognosis) Level 1.

Does this intervention help? (Treatment Benefits) Level 1.

What are the COMMON harms? (Treatment Harms) Level 1.

What are the RARE harms? (Treatment Harms) Level 1.

Is this (early detection) test worthwhile? (Screening) Level 1I.

## Highlights

• We searched PubMed, Embase and Cochrane Library databases.
• This study included 22 randomized controlled trials with a total of 2354 patients.
• Acoustic therapy combined with cognitive behavior can treat chronic tinnitus.

## Introduction

Tinnitus is a subjective auditory experience occurring in the absence of external auditory stimuli. It is not considered as a separate disease entity.^1^ Tinnitus is most often experienced as involuntary phantom hearing. It is caused by abnormal neural activity, mediated by the pathological stimulation of the organs that sense sound or their associated conduction pathways. Hearing loss frequently accompanies tinnitus.^2^, ^3^ Tinnitus affects approximately 10% to 15% of the adults worldwide, with a significantly higher prevalence in men than in women.^4^, ^5^ In Asia, the overall prevalence of tinnitus is even higher at 18.6%, and approximately 11.9% in adults aged from 45 to 79 years.^6^ No large-scale epidemiologic study of tinnitus has been conducted in China; approximately 10% of the population has experienced tinnitus, whereas only 5% has received professional medical advice or treatment.^7^

High prevalence of tinnitus and its associated socio-economic burden, particularly among elderly adults and in economically developed regions, highlight the need and necessity for in-depth research. Factors associated with the tinnitus and its severity principally encompass hearing loss, prolonged exposure to high-decibel environments (either at work or in the workplace), and overall health status.^8^, ^9^ Furthermore, tinnitus has also been associated with cardiovascular problems, a history of substance use, ear infections or inflammation of the ear (e.g., otitis media), head and neck injuries, thyroid dysfunction, Meniere’s disease, otosclerosis, sudden deafness, and vestibular nerve sheath tumors.^10^, ^11^, ^12^

Numerous non-invasive strategies facilitate tinnitus treatment, including cognitive behavioral therapy,^13^ transcranial magnetic stimulation,^14^, ^15^ electrical stimulation,^16^ sound therapy,^17^ and tinnitus retraining therapy.^18^ Additionally, innovative treatment modalities, such as acceptance and commitment therapy and virtual reality therapy have emerged, which provide diversified options for tinnitus treatment.

Cognitive behavioral therapy is considered the gold standard of psychotherapy.^12^, ^19^, ^20^ In contrast, sound therapy and tinnitus retraining therapy alleviate tinnitus symptoms by promoting neuroplasticity in the auditory system.^21^ Acceptance and commitment therapy and virtual reality therapy offer novel and innovative approaches to the treatment of tinnitus. However, evidence of their relative effectiveness is limited. Therefore, we aimed to assess the effectiveness and feasibility of these Non-Invasive Treatments (NITs) for improving chronic tinnitus symptoms through a systematic review and Network Meta-Analysis (NMA).

## Methods

### Search strategy

According to the Preferred Reporting Items for Systematic Reviews and Meta-Analyses reporting guidelines,^16^, ^17^ the PubMed, Embase and Cochrane Library databases were searched from data construction to December 31, 2022. The search was performed using a combination of subject terms and free words with the following search terms: Take PubMed as an example; the search strategy is as follows: “Tinnitus” [Title/Abstract] AND (“Cognitive Behavioral Therapy” OR “Transcranial Magnetic Stimulation” OR “Transcranial Direct Current Stimulation” OR “Percutaneous Electrical Nerve Stimulation” OR “Tinnitus Retraining Therapy” OR “Sound Therapy” OR “Maskers” OR “Music Therapy” OR “Virtual Reality” OR “Acceptance and Commitment Therapy” OR “Placebo” OR “Psychoeducation” OR “Counseling” OR “Online Discussion” OR “Relaxation”). The search language was limited to English.

### Inclusion and exclusion criteria

#### Inclusion criteria

The inclusion criteria were developed according to the Population, Intervention, Comparison, Outcomes and Study (PICOS) principles as follows: 1) Type of study: Randomized Controlled Trials (RCTs); 2) Subjects: adults (≥18 years of age) suffering from chronic tinnitus (lasting at least 3 months); 3) Interventions: Acceptance and commitment therapy (A), Cognitive behavioral therapy (C), Sound therapy (S), Transcranial magnetism therapy (T), Electrical stimulation (E), Virtual reality therapy (V), Tinnitus Retraining therapy (R), General psychotherapy (D), and Placebo (P); 4) Outcome indicators: ① Tinnitus Handicap Inventory (THI), ② Tinnitus Questionnaire (TQ), ③ Hospital Anxiety and Depression Scale (Hospital Anxiety and Depression Scale-Anxiety-Depression (HADS-D), ④ Insomnia Severity Index (ISI), ⑤ Visual Analogue Scales-Loudness (VAS-L), and ⑥ Visual Analogue Scales-Distress (VAS-D).

#### Exclusion criteria

The exclusion criteria were as follows: 1) Case reports, book chapters, review articles, expert opinions, and so on.; 2) Studies on other ear or hearing problems (e.g., middle ear infections and sudden deafness); 3) Studies with insufficient or publicly unavailable data; 4) Studies not published in English; and 5) Studies with only abstracts were available or unavailable full text.

### Literature screening and data extraction

To ensure the accuracy and dependability of the analysis, the literature screening and data extraction were conducted by two researchers independently. The screening and extraction process followed strict predefined criteria and involved cross-checking of information; in the case of disagreement, they reached a consensus would be reached through academic discussion or third-party arbitration. Data extraction included, but was not limited to, basic information about the included studies (e.g., the authors, year of publication, study design, and sample size), baseline characteristics of the study population (e.g., age and sex, among others.), and interventions undertaken.

### Literature quality evaluation

Two investigators (TT Lu and QX Wang) independently evaluated the risk of bias of in the included studies independent using the RCTs Risk of Bias Evaluation Tool. The results were cross-checked to ensure consistency. In the event of disagreement between TT Lu and QX Wang during the risk of bias and quality assessment process, a third researcher (ZJ Yan) will arbitrated to ensure the fairness and accuracy of the evaluation.

### Statistical analysis

Stata 14.0 software was used for NMA, continuous variables, and Standardized Mean Differences (SMDs) and their 95% Confidence Intervals (95% CI) were calculated; For dichotomous variables, the Odds Ratio (OR) and its 95% CI were calculated. The I^2^ statistic was used to assess statistical heterogeneity. A fixed-effect model was used for I^2^ < 50% and *p* > 0.01, else a random-effects model was used. To identify potential publication bias and small sample effects, funnel plots were used for visual assessment. Each treatment outcome was compared quantitatively by a Surface Under the Cumulative Ranking curve (SUCRA). Treatments with higher SUCRA values indicated a higher possible efficacy. To enhance the robustness of our results, data consistency or inconsistency was assessed, with a statistically significant difference of bilateral *p* < 0.05.

## Results

### Literature screening process and results

**Figure 1:**
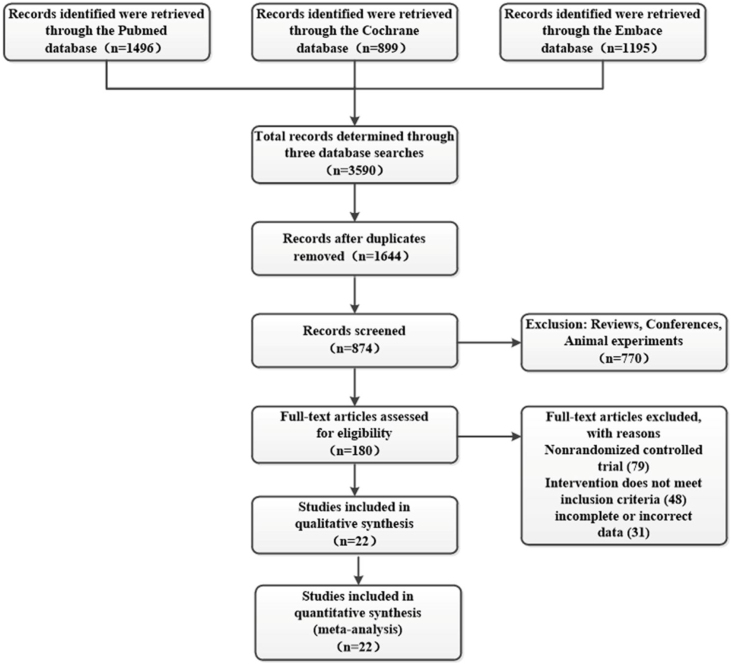
Schematic diagram of the literature screening process and results.

After the initial search, a total of 3590 relevant articles were retrieved. After removing 1,946 duplicate articles; 1,644 articles were screened for their titles and abstracts. A total of 180 articles met the inclusion criteria. Full-text screening resulted in the exclusion of 158 articles, primarily because of inconsistent study design, data extraction, or no control group. Finally, 22 articles were included in this systematic review and NMA (Fig. 1).

### Basic characteristics and risk of bias analysis of included studies

**Figure 2:**
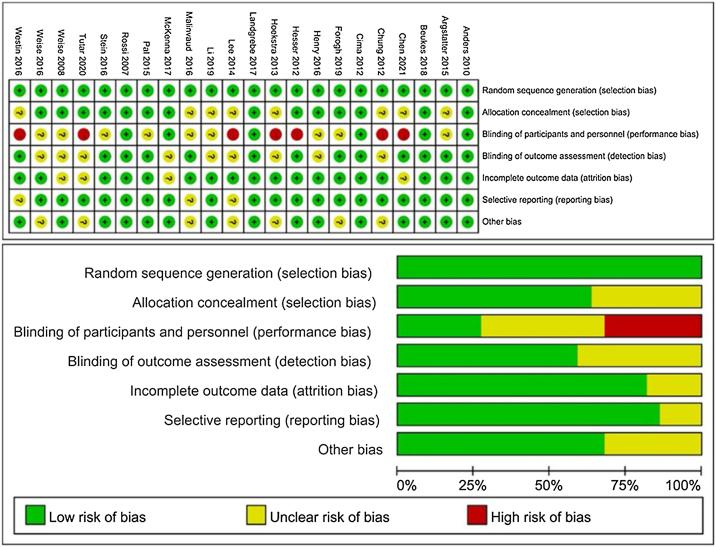
Results of the risk of bias evaluation of the included literature.

The 22 studies included in this systematic review and NMA,^13^, ^14^, ^15^, ^16^, ^17^, ^18^, ^19^, ^20^, ^21^, ^22^, ^23^, ^24^, ^25^, ^26^, ^27^, ^28^, ^29^, ^30^, ^31^, ^32^, ^33^, ^34^ comprised 2354 patients with chronic tinnitus, The studies included numerous NITs (Table 1). Assessment of the risk of bias results based on the Risk of Bias Evaluation Tool for RCTs recommended by the Cochrane Handbook 5.1.0 suggested that all the studies were at risk of bias in terms of randomized sequence generation (100%), most matched concealment (63.6%), blinded assessment (outcome evaluation, 59.1%), data incompleteness (81.8%), selective reporting bias (86.4), and other bias (68.2%). Few studies blinded to the assessment (participants and performers, 27.3%) were rated at a low risk of bias in line with our requirements (Fig. 2).

**Table 1:** General information on the included literature.

Author	Year	Country	Research type	Sample size		Average age (years) Mean (SD)		Male sex ratio (%)		Intervention measure		Outcome index
				Experimental group	Control group	Experimental group	Control group	Experimental group	Control group	Experimental group	Control group	
Westin^14^	2016	Germany	RCT	62	62	47.81 (12.26)	47.51 (14.07)	59.7	59.7	C	D	①②③④
Cima^23^	2012	Britain	RCT	247	245	54.63 (12.02)	53.74 (11.05)	61	65	C	D	①②
Li^24^	2019	China	RCT	50	50	42.66 (3.72)	43.81 (4.12)	56	62	C	S	①
Hesser^15^	2012	Germany	RCT	32/35	32	48.8 (13.4)/ 50.1 (16.4)	48.4 (14.2)	43.8/42.9	43.4	C	A/D	①③④
Beukes^16^	2018	Britain	RCT	46	46	50.65 (12.19)	55.26 (11.62)	63	57	C	D	①④
Chung^25^	2012	America	RCT	10	12	/	/	/	/	T	P	①②
Landgrebe^17^	2017	Germany	RCT	71	75	48.1 (12.5)	49.9(13.2)	76	68	T	P	①②
Hoekstra^26^	2013	NZ	RCT	26	24	50 (12)	55 (12)	100	63	T	P	①②⑥
Rossi^27^	2007	Italy	RCT	22	20	/	/	/	/	T	P	①②⑥
Pal ^28^	2015	NZ	RCT	21	21	48.0 (9.9)	51.6 (12.2)	57.1	57.1	E	P	①⑤⑥
Forogh^21^	2019	Ireland	RCT	11	11	49.81 (4.14)	46.63 (5.26)	36.4	36.4	E	P	①⑤⑥
Tutar^20^	2020	Turkey	RCT	20	20	/	/	/	/	E	P	①
Lee^29^	2014	Korea	RCT	45	20	46.6 (13.9)	46.6 (13.9)	42.2	28	E	P	①⑤⑥
Henry^18^	2016	America	RCT	42/34	39/33	62.4 (9.8)/ 60.1 (10.1)	62.7 (10.6)/ 61.2 (8.8)	95.2/97.1	100/97	R/D	S/P	①
Westin^19^	2011	Sweden	RCT	20	20/22	53.5 (12.84)	48.9 (14.5)/ 49.5 (11.86)	64	40/36	R	A/AP	①③④
Chen^30^	2012	China	RCT	30	30	37 (20.50)	45 (18.25)	36.7	50	S	D	①⑤
Malinvaud^22^	2016	France	RCT	55	61	49.14(12.11)	52.2 (12.64)	65.45	80.33	C	V	①③⑤
McKenna^33^	2017	America	RCT	45	44	53 (14)	47 (17)	75.5	36.4	C	D	①③⑤
Weise^35^	2008	Germany	RCT	52	59	49.46 (11.83)	52.93 (11.92)	55.8	55.9	C	P	①⑤⑥
Argstatter^32^	2015	Germany	RCT	146	144	45.1 (12.4)	53.2 (12.0)	71.2	65.3	S	D	①
Stein^34^	2016	Germany	RCT	50	50	47.68 (9.94)	47.13 (11.70)	66	68	S	P	①⑤
Anders^31^	2010	Czech Republic	RCT	20	22	27∼66	20∼69	/	/	S	D	

(1) Interventions: Acceptance and Commitment Therapy (A), Cognitive Behavioral Therapy (C), Sound Therapy (S), Transcranial Magnetism Therapy (T), Electrical Stimulation (E), Virtual Reality Therapy (V), Tinnitus Retraining Therapy (R), General Psychotherapy (D), and Placebo (P).

(2) Outcome Indicators: ① Tinnitus Handicap Index (THI), ② Tinnitus Questionnaire (TQ), ③ Hospital Anxiety and Depression Scale (HADS-D), ④ Insomnia Severity Index (ISI), ⑤ Visual Analog Scale-Loudness (VAS-L), ⑥ Visual Analog Scale-Distress (VAS-D).

### Net meta-analysis

#### Tinnitus disability inventory (THI)

**Figure 3:**
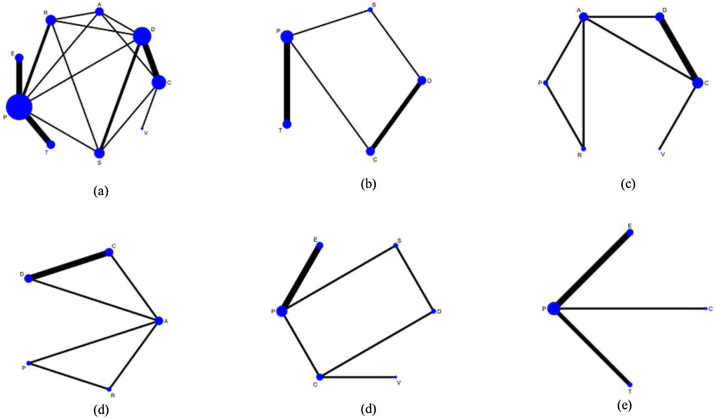
Network evidence maps. (a) THI; (b) TQ; (c) HADS-D; (d) ISI; (e) VAS-L; (f) VAS-D. A, Acceptance and Commitment Therapy; C, Cognitive Behavioral Therapy; S, Sound Therapy; T, Transcranial Magnetic Therapy; E, Electrical Stimulation Therapy; V, Virtual Reality Therapy; R, Tinnitus Retraining Therapy; D, General Psychotherapy; P, Placebo.

**Figure 4:**
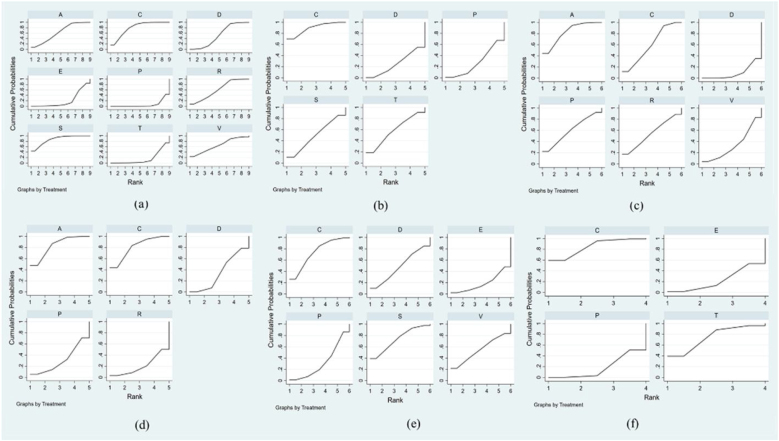
SUCTA ordering diagram. (a) THI; (b) TQ; (c) HADS-D; (d) ISI; (e) VAS-L; (f) VAS-D. A, Acceptance and Commitment Therapy; C, Cognitive Behavioral Therapy; S, Sound Therapy; T, Transcranial Magnetic Therapy; E, Electrical Stimulation Therapy; V, Virtual Reality Therapy; R, Tinnitus Retraining Therapy; D, General Psychotherapy; P, Placebo.

**Figure 5:**
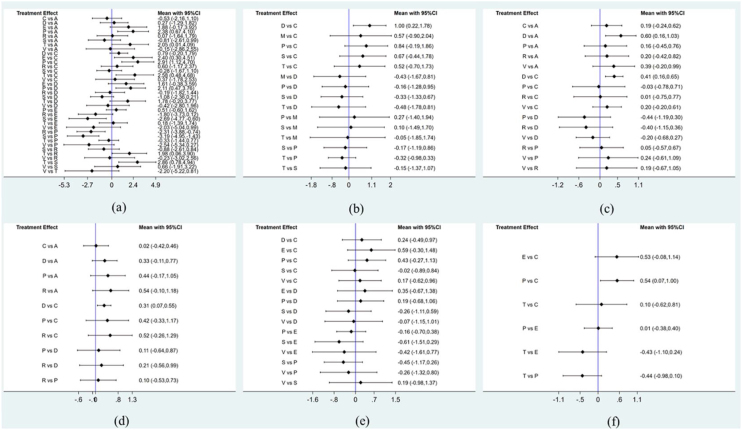
Forest plots for direct/indirect comparisons. (a) THI; (b) TQ; (c) HADS-D; (d) ISI; (e) VAS-L; (f) VAS-D. A, Acceptance and Commitment Therapy; C, Cognitive Behavioral Therapy; S, Sound Therapy; T, Transcranial Magnetic Therapy; E, Electrical Stimulation Therapy; V, Virtual Reality Therapy; R, Tinnitus Retraining Therapy; D, General Psychotherapy; P, Placebo.

Seventeen studies addressed nine chronic tinnitus treatments and their effects on the THI scores. Fig. 3a illustrates the network evidence map. According to the SUCRA ranking chart, the effects of various treatments on THI scores, in descending order, were as follows: Sound Therapy (86.9%), Cognitive Behavioral Therapy (79.8%), Virtual Reality (65.0%), Acceptance and Commitment Therapy (62.4%), Tinnitus Retraining Therapy (60.7%), General Psychotherapy (51.9%), Electrical Stimulation (20.7%), Transcranial Magnetic Stimulation (16.3%), and Placebo (6.5%), as shown in Fig. 4a. The direct or indirect comparison results showed that the most significant difference in effectiveness between sound therapy and electrical stimulation was the most significant, with an effect size of −2.69 (95% CI −4.77, −0.60) (Fig. 5a).

#### Tinnitus questionnaire (TQ)

Ten studies addressed the effectiveness of five chronic tinnitus treatments regarding the TQ. Fig. 3b illustrates the network evidence map. According to the SUCRA ranking chart, the effectiveness of the various treatments in terms of TQ, in descending order, was as follows: cognitive behavioral therapy (89.5%), transcranial magnetic stimulation (58.3%), sound therapy (49.6%), placebo (27.2%), and general psychotherapy (25.4%) (Fig. 4b). Results of the indirect or direct comparisons suggested the most significant difference in effect sizes between general psychotherapy and cognitive behavioral therapy, with an effect size of 1.00 (95% CI 0.22, 1.78) (Fig. 5b).

#### Hospital anxiety and depression scale (HADS-D)

Five studies reported on changes in the HADS-D scores for six chronic tinnitus treatments. Fig. 3c illustrates the network evidence. According to the SUCRA ranking chart, the effectiveness of different treatments on HADS-D scores, in descending order, was as follows: tinnitus retraining therapy (R, 54.4%), virtual reality therapy (V, 33.7%), and general psychotherapy (D, 9.47%). Fig. 4c illustrates the effect of different treatments on the HADS-D score. The direct or indirect comparison results suggested the most significant difference in HADS-D scores between general psychotherapy and acceptance and commitment therapy, with an effect size of 0.60 (95% CI 0.16, 1.03) (Fig. 5c).

#### Insomnia severity index (ISI)

Four studies reported on changes in the ISI score for five chronic tinnitus treatments. Fig. 3d illustrates the network evidence. According to the SUCRA ranking chart, the effectiveness of different treatments on ISI scores, in descending order, was as follows: Acceptance and commitment therapy (A, 83.2%), Cognitive behavioral therapy (C, 80.5%), general psychotherapy (D, 34.9%), placebo (P, 30.8%), and tinnitus retraining therapy (R, 20.6%) (Fig. 4d) Results of direct or indirect comparisons showed the most significant difference in effect size regarding ISI scores between general psychotherapy and cognitive behavioral therapy, with a specific effect size of 0.31 (95% CI 0.07, 0.55) (Fig. 5d).

#### Visual analog scale-loudness (VAS-L)

Eight studies reported on changes in the VAS-L scores for six chronic tinnitus treatments. Fig. 3e illustrates the network evidence. According to the SUCRA ranking chart, the effectiveness of different treatments on VAS-L scores, in descending order, was as follows: sound therapy (S, 73.5%), cognitive behavioral therapy (C, 73.4%), virtual reality therapy (V, 54.6%), general psychotherapy (D, 48.1%), placebo (P, 31.6%), and electrical stimulation therapy (E, 18.9%) (Fig. 4e). The direct or indirect methods could not be compared (Fig. 5e).

#### Visual analog scale-distress (VAS-D)

Six studies reported on changes in the VAS-L scores for four chronic tinnitus treatments. Fig. 3f illustrates the network evidence. The effects of the different treatments on the VAS-D were, in descending order: Cognitive Behavioral Therapy (C, 84.7%), Transcranial Magnetic Therapy (T, 74.6%), Electrical Stimulation Therapy (E, 22.5%), and Placebo (P, 18.1%), as detailed in Fig. 4f. Particularly note that the effect size between Placebo and Cognitive Behavioral Therapy was 0.54 with a confidence interval of (0.07, 1.00) (Fig. 5f).

### Sensitivity analysis and publication bias evaluation

**Figure 6:**
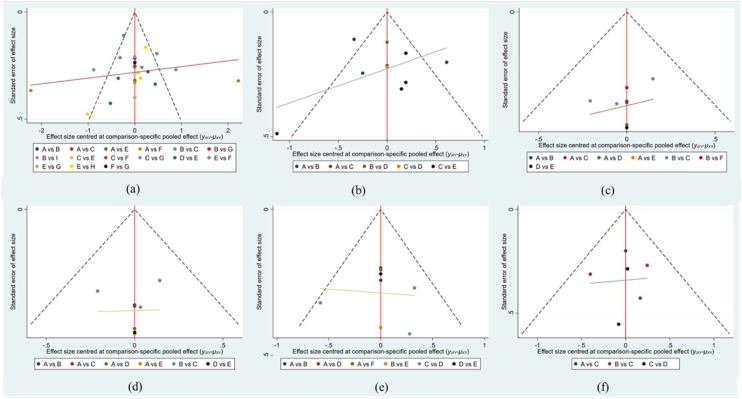
Funnel plot of publication bias. (a) THI; (b) TQ; (c) HADS-D; (d) ISI; (e) VAS-L; (f) VAS-D. A, Acceptance and Commitment Therapy; C, Cognitive Behavioral Therapy; S, Sound Therapy; T, Transcranial Magnetic Therapy; E, Electrical Stimulation Therapy; V, Virtual Reality Therapy; R, Tinnitus Retraining Therapy; D, General Psychotherapy; P, Placebo.

To verify the robustness and reliability of our results of this study, sensitivity analyses were conducted using a hair-by-hair approach to examine its effect on the overall findings. The results showed that the combined effect sizes and SUCRA rankings remained stable in all sensitivity analyses, implying that the results of this study have high reliability. We used Begg’s funnel plot to assess possible publication bias; although few studies were outside the funnel, we attained good symmetry, suggesting marginal publication bias among the included studies (Fig. 6).

## Discussion

NITs have gradually received widespread attention from the medical community in the clinical management of chronic tinnitus, Several clinical practice reports have demonstrated a growing trend of NIT use.^35^, ^36^ Nevertheless, evidence supporting NITs with optimal efficacy and safety in the management of chronic tinnitus is limited. Traditional meta-analyses are usually limited to two-by-two comparisons, which require more comprehensiveness in the case of multiple treatment options. As a result, it is difficult for traditional methods to provide a comprehensive and conclusive evidence-based medical basis. To address this issue, we conducted an NMA method based on the Bayesian framework^37^ to compare the efficacy and safety of different NITs for the treatment. We synthesized direct and indirect comparisons and provided a more plausible evidence-based medical rationale for the clinical treatment of chronic tinnitus.

We comprehensively evaluated various chronic tinnitus treatments on multiple clinical metrics through NMA, filling the gap of established studies in this area. Unlike previous studies, this study not only quantified the performance of multiple treatments under different assessment criteria, but also further clarified the strengths of each treatment in specific areas. When using the THI as an assessment criterion, sound therapy took a clear lead with 86.9% effectiveness. This data confirm not only the superiority of sound therapy in alleviating tinnitus-induced dysfunction, but also the highly significant difference in THI scores with the least effective, electrical stimulation,^21^ Electrical stimulation reduces tinnitus loudness by decreasing auditory gain, which can be modulated by prolonging the reduction or enhancement of background sound and the enhancement of auditory gain. This in turn can partially explain tinnitus and hyperacusis onset and the maintenance mechanisms.^38^ Furthermore, sound therapy can “mask” or “blend” tinnitus sounds by increasing loudness tolerance^39^ and reducing auditory hypersensitivity,^40^ thereby reducing their interference and effectively modulating the patient’s perception of tinnitus, leading to more functional improvements,^29^ which is also consistent with the previous findings.^21^, ^29^ We observed significant differences in THI scores between sound therapy and electrical stimulation, emphasizing the importance of sound therapy in treating chronicity. Regarding TQ, cognitive behavioral therapy, including psychoeducation, relaxation, exposure techniques, and behavioral responses, is usually combined with positive thinking training,^22^ Cognitive behavioral therapy demonstrated a high effect size of 89.5%, showcasing superiority. By contrast, previous studies did not differentiate between the treatments based on different assessment criteria.^13^, ^14^, ^22^, ^23^ For patients who are highly disturbed by tinnitus, mainly if the symptoms result in a significant psychological burden, Cognitive Behavioral therapy is not only reduce the subjective distress of tinnitus but also improves the overall quality of life of the patients experiencing psychological burdens.^14^, ^23^

We divided the performance of NITs under different psychological and physiological assessment criteria, which have not been discussed previously.^14^, ^15^, ^16^, ^24^, ^25^, ^26^ Especially in the assessment dimensions HADS-D and ISI, we found that Tinnitus Retraining Therapy, Virtual Reality therapy, and Acceptance and Commitment Therapy were superior under specific conditions. Tinnitus Retraining Therapy is a treatment for tinnitus and decreased sound tolerance. Soma topoietic.^41^, ^42^ Ear virtual reality therapy can provide users with a sense of presence and immersion through 360° visual displays, spatial acoustics, and haptic feedback to induce changes at the brain level to alleviate tinnitus.^43^ Our results also showed that on HADS-D, Tinnitus Retraining Therapy and Virtual Reality therapy demonstrated their potential in alleviating anxiety and depression triggered by tinnitus with 54.4% and 33.7% effectiveness. On ISI, Acceptance and Commitment Therapy and Cognitive Behavioral Therapy topped the list with 83.2% and 80.5% effectiveness, which may imply that these two treatments involve specific neural pathways or psychological mechanisms, suggesting that these two psychotherapeutic treatments may be the most effective options in dealing with insomnia due to tinnitus, especially Acceptance and Commitment Therapy, which may be able to help the patient to deal with insomnia by teaching them how to accept and cope with tinnitus in a accept and cope with tinnitus in a more constructive way, while achieving improved sleep quality.^14^, ^18^ Meanwhile on the VAS-L and VAS-D, Sound Therapy and Cognitive Behavioral Therapies were almost equal, each slightly ahead on a different scale. Tinnitus is usually not associated with labyrinthine disorders, auditory neuritis, or other organic disorders, but rather is considered an unexplained somatic symptom, i.e., a diagnosis of a somatic disorder is insufficient to explain these symptoms.^44^ Cognitive Behavioral Therapy was developed in the early 1960s to guide patients in replacing dysfunctional thinking, unhealthy behaviors, and unrealistic cognitive assessments with more realistic and adaptive assessments. Patients with tinnitus frequently experience higher levels of anxiety and depression and often exhibit chorea, impulsivity, self-destructive and heterosexually destructive behaviors.^45^ Thus, tinnitus appears to coexist with various forms of psychopathology, and thus, Cognitive Behavioral Therapy figures prominently in its effectiveness. It has also been shown that cognitive-behavioral therapy significantly reduces tinnitus-induced anxiety and malaise within three months of treatment.^46^, ^47^ Our results further confirm that both Sound therapy and Cognitive Behavioral Therapy are highly effective in dealing with tinnitus-induced discomfort and distress.

Transcranial magnetic stimulation is a non-invasive neuromodulation technique for cerebral hyperexcitability disorders aimed at reducing neural activity in non-auditory regions associated with the pathogenesis of tinnitus.^48^, ^49^ It positively affects the metabolic activity of cranial neurons and improves tinnitus symptoms. Electrical stimulation therapy is a method of altering somatosensory inputs non-invasively through the use of electrical stimulation devices. This method generates somatosensory stimulation, leading to abnormal activation of the dorsal cochlear nucleus. This activation affects the physiological correlates of tinnitus exerting a role in alleviating tinnitus,^50^, ^51^ however, in our results, Transcranial Stimulation Method and Electrical Stimulation Therapy were not outstanding in treating tinnitus among the many non-invasive therapeutic methods.

We conducted an NMA to comprehensively assess numerous NITs for chronic tinnitus, thereby providing informative value for clinical practice. Nonetheless, some limitations should be considered while interpreting these results. First, the literature partially introduced a risk of bias regarding blinding. Second, variations in the patient age, medication dose, and course of treatment increased clinical heterogeneity and made comparability among the studies less likely. Third, the small sample size of the included literature may have led to sampling bias, which can affect the reliability of our results. Therefore, future studies should include more standardized, high-quality, large-sample and multicenter randomized controlled double-blinded RCTs to provide a stronger scientific basis for the safety and efficacy of NITs for treating chronic tinnitus.

## Conclusions

Upon combining multiple clinical outcome indicators (THI, TQ, HADS-D, ISI, VAS-L, and VAS-D), Sound Therapy, Cognitive Behavioral Therapy, and Acceptance and Commitment Therapy demonstrated relatively high effectiveness in chronic tinnitus treatment. Particularly, sound therapy, cognitive behavioral therapy, and acceptance and commitment therapy demonstrated the highest effectiveness for THI and VAS-L scores, TQ and VAS-D scores, and HADS-D and ISI scores, respectively.

## Authors’ contributions

Tingting Lu: Conceptualization; data curation; methodology; software; visualization; writing-original draft.

Zhaojun Yan: Resources; software; project administration; writing-review & editing.

Qingxin Wang: Data curation; supervision; validation; visualization.

Ziyan Gu: Resources, software; visualization.

Zefang Li: Resources; software; visualization.

## Funding

This research did not receive any specific grant from funding agencies in the public, commercial, or not-for-profit sectors.

## Data availability statement

The data are available from the corresponding author on reasonable request.

## Conflicts of interest

The authors declare no conflicts of interest.
